# “AI’s gonna have an impact on everything in society, so it has to have an impact on public health”: a fundamental qualitative descriptive study of the implications of artificial intelligence for public health

**DOI:** 10.1186/s12889-020-10030-x

**Published:** 2021-01-06

**Authors:** Jason D. Morgenstern, Laura C. Rosella, Mark J. Daley, Vivek Goel, Holger J. Schünemann, Thomas Piggott

**Affiliations:** 1 Department of Health Research Methods, Evidence, and Impact, McMaster University, Hamilton, Ontario Canada; 2 Dalla Lana School of Public Health, University of Toronto, Toronto, Ontario Canada; 3 Institute for Clinical Evaluative Sciences, Toronto, Ontario Canada; 4 Public Health Ontario, Toronto, Ontario Canada; 5 Vector Institute, Toronto, Ontario Canada; 6 Department of Computer Science, Western University, London, Ontario Canada; 7 Department of Biology, Western University, London, Ontario Canada; 8 Department of Actuarial Sciences and Statistics, Western University, London, Ontario Canada; 9 Brain and Mind Institute, Western University, London, Ontario Canada; 10 Department of Medicine, McMaster University, Hamilton, Ontario Canada

**Keywords:** Population health, Preventive medicine, Machine learning, Community medicine, Big data, Epidemiology, Qualitative

## Abstract

**Background:**

Our objective was to determine the impacts of artificial intelligence (AI) on public health practice.

**Methods:**

We used a fundamental qualitative descriptive study design, enrolling 15 experts in public health and AI from June 2018 until July 2019 who worked in North America and Asia. We conducted in-depth semi-structured interviews, iteratively coded the resulting transcripts, and analyzed the results thematically.

**Results:**

We developed 137 codes, from which nine themes emerged. The themes included opportunities such as leveraging big data and improving interventions; barriers to adoption such as confusion regarding AI’s applicability, limited capacity, and poor data quality; and risks such as propagation of bias, exacerbation of inequity, hype, and poor regulation.

**Conclusions:**

Experts are cautiously optimistic about AI’s impacts on public health practice, particularly for improving disease surveillance. However, they perceived substantial barriers, such as a lack of available expertise, and risks, including inadequate regulation. Therefore, investment and research into AI for public health practice would likely be beneficial. However, increased access to high-quality data, research and education regarding the limitations of AI, and development of rigorous regulation are necessary to realize these benefits.

**Supplementary Information:**

The online version contains supplementary material available at 10.1186/s12889-020-10030-x.

## Background

After more than 60 years of evolution as a field [1], artificial intelligence (AI) has become ubiquitous in the last decade. These changes have prompted both excitement and trepidation regarding potential impacts in virtually all human endeavours, including public health. Interestingly, despite widespread discussion, there is no universally accepted definition of AI. One definition, offered by Kaplan and Haenlein, describes AI as “a system’s ability to interpret external data correctly, to learn from such data, and to use those learnings to achieve specific goals and tasks through flexible adaptation.” [2] Cybernetics researchers developed some of the first AI systems, including designs for an artificial neuron in 1943 [1]. The AI-term was adopted by the field following a workshop in 1956 [1]. In the ensuing years, AI research went through several boom and bust cycles, including a focus on rules-based expert systems in the 1970s and -80s [3]. In the early 2000s, increasing computational power, the ability to record and access vast amounts of data, and several enabling theoretical developments encouraged a renewed focus on data-driven approaches to AI [1]. Many of these approaches fall under the subfield of machine learning, which can be loosely defined as a “field of study that gives computers the ability to learn without being explicitly programmed.” [4] Machine learning forms the foundation for most modern applications of AI, including targeted online advertising, conversational AI-assistants, and movie recommendations. These approaches are data-hungry, often relying on big data, or information flows with abundant volume, velocity, and variety [5].

Beyond the most visible applications of AI among the tech giants of Silicon Valley, it has started to infiltrate healthcare and public health. In healthcare, AI applications have been reported that match or outperform physicians in various domains including radiology [6], dermatology [7], and pathology [8]. Additionally, some hospitals have begun to integrate AI into the clinical workflow, as in the case of New York University Langone Health’s predictive analytics unit [9]. While considerable attention has been paid to AI in healthcare, there has been less attention on its impact in public health [10]. Despite this, public health researchers and practitioners have begun applying AI to diverse projects such as scanning the internet for nascent outbreaks [11], predicting suicide using electronic health records [12], and identifying risk factors [13]. As such, there has been growing optimism regarding the potential for AI to improve public health [14]; however, few AI systems have actually been implemented within public health organizations. Moving forward, there are serious concerns regarding AI’s impacts on privacy, interpretability, and potential for bias [15, 16]. Also, there has been criticism that AI as applied in precision public health is merely scaling up the precision medicine approach [17]. The potential to move beyond biomedical applications of AI to models incorporating rich characterizations of the social determinants of health has been identified as a promising and largely unexplored frontier. A clearer understanding of AI’s relevance to public health, which is presently absent from the literature, is needed to navigate the opportunities and risks. We conducted an initial step towards filling this gap by examining the perspectives of experts in public health and AI regarding implications of AI for public health practice.

## Methods

We used a fundamental qualitative descriptive study design [18] to explore the impacts of AI on public health practice (see Additional file 1 for the consolidated criteria for reporting qualitative research (COREQ) checklist). We conducted in-depth semi-structured interviews with experts in public health and/or artificial intelligence. We selected participants using a mixed sampling strategy. A thematic analysis underlies our conclusions. The project was approved by the Hamilton Integrated Research Ethics Board (Project #: 4825).

### Participant recruitment

We sampled contributors from a pool of experts in public health and artificial intelligence using a combination of convenience, snowball, and stratified purposeful sampling [19]. This included identifying participants known to us, known to interviewees, and performing internet searches. We selected prospective interviewees to represent a range of perspectives, including academia, government, industry, and non-governmental organizations (NGOs); a range of disciplines, including public health physicians, epidemiologists, computer scientists, and policy-experts; and a range of contexts, including high-income and low-income countries.

We initially contacted all participants with a standard email invitation. If we did not receive a response, we sent a single follow-up email after 1 week but made no further efforts to contact prospective participants. We answered interested invitees’ questions and emailed them a consent form. We received written consent to participate.

### Semi-structured interviews

We developed an interview guide to support the semi-structured interview process, which was pilot tested (see Additional file 2 for interview guide). This guide was based on the essential public health functions developed by the Pan-American Health Organization [20]. Functions include examples such as “monitoring, evaluation, and analysis of health status”, “health promotion”, and “research in public health”. We framed interview questions around AI’s impact on essential public health functions, as well as several current issues in public health. In addition to pre-specified interview questions, we followed up on statements made by interviewees [21]. Also, we focused on domains most related to the interviewee’s expertise. Therefore, we asked different questions with each interviewee.

At the beginning of each interview we discussed the consent form, answered questions, and obtained and archived a signed copy of the form (see Additional file 3 for a description of the interviewers). No one except for the interviewer(s) and interviewee were present. We conducted interviews once with each participant either over the phone or in-person (depending on the interviewee’s preference) and recorded them. We stored the recordings in an encrypted, password-protected folder. Field notes were made during and after interviews.

We conducted fifteen interviews, which is consistent with the number collected in comparable studies [21]. Additionally, we verified that fewer new codes were generated in later interviews, indicating relative saturation (see results). However, given the broad area of inquiry, absolute saturation could not be guaranteed. We transcribed the interviews using Descript [22], an automated transcription program. Then, we manually edited and de-identified transcripts. Transcripts were only viewed by the researchers.

### Coding and thematic analysis

Following transcription and de-identification, we imported transcripts into an encrypted project within Dedoose [23], a mixed-methods research data-processing program. Then, two investigators iteratively created codes used to categorize components of each transcript. Following initial coding, we conducted a three-stage process to generate themes. First, two investigators independently identified five initial themes, which were compared. Then, we identified and discussed topics and themes from each interview. Lastly, we identified the most-used codes and iteratively combined them into themes. We collated the results of each stage into the list of themes.

Following initial thematic analysis, we re-organized and condensed the codes. Additionally, we reviewed the transcripts to identify key quotes related to identified themes. Throughout this process, we continued to refine themes until reaching consensus. Participants did not provide feedback on findings.

### Word cloud

We built a word cloud of the study codes using WordArt [24] software. The number of applications of each code determined its size in the word cloud. The word cloud excludes the most general root-codes.

## Results

Of the 25 interview invitees, 7 (28%) did not respond and 3 (12%) declined to participate. In all cases when the interview was declined, the reason given was that prospective participants were overwhelmed with their responsibilities. The fifteen conducted interviews had a median duration of 45 min.

Table 1 shows the participants’ characteristics. Approximately one quarter of the participants were female. Most respondents’ primary expertise was in epidemiology, medicine, or public health; however, all had significant experience or interest in AI. Participants differed widely in career stage and age.

**Table 1:** Participant characteristics

	Number of Participants (%)
**Female**	4 (26.7%)
**Continent**	
Asia	1 (6.7%)
North America	14 (93.3%)
**Organization Type (not mutually exclusive)**	
Academia	10 (66.7%)
Government	8 (53.3%)
Industry	3 (20%)
Non-governmental Organization	2 (13.3%)
**Primary Area of Expertise**	
Artificial Intelligence	2 (13.3%)
Epidemiology	3 (20%)
Medicine	3 (20%)
Public Health	7 (46.7%)

**Fig. 1:**
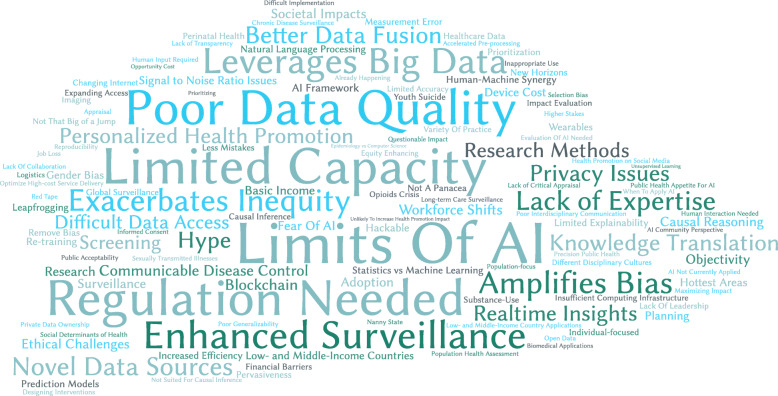
A word cloud of the study codes, excluding the most general root-codes. The size of each code reflects the number of times that it was applied.

We developed 135 codes, which were applied 572 times to 377 excerpts. The number of new codes generated decreased over time, with interviews 13, 14, and 15 producing three, two, and zero new codes respectively. The coding system included 3 levels of nested codes, with most in the second level. The major root-codes were Opportunities, Barriers, Threats, and Hype. Including sub-codes, we used the Opportunities root-code the most, a total of 188 times. We applied Barrier codes 126 times and Threats codes 62 times. See Fig. 1 for a word cloud of the codes used and Additional file 4 for a table containing detailed coding information.

### Thematic analysis

Nine themes emerged (See Table 2). A description of each theme, including key quotes, follows.

**Table 2:** Themes identified

Opportunities Themes	Barriers Themes	Risks Themes
• From Big Data to Big Insights • AI Will Improve Public Health Interventions	• What is AI for? • Limited Capacity • Lack of Quality Data	• Bias Must be Controlled • Uncertain Impact on Inequity • Hold the Hype • Rigorous Regulation Required

### From big data to big insights

The potential for AI to convert big data into actionable public health insights was a major focus of discussion. Many experts emphasized that public health has always been very data-driven, but that AI could expand these activities even further.

The broad implications of AI for public health are that AI can serve as an intermediary between the huge amounts of data that we generate and action.

[Participant ID # 4] (see Additional file 5 for characteristics associated with each participant ID).

Specifically, interviewees highlighted surveillance as a top domain of public health for AI applications (see Additional file 6 for further supporting quotations). AI’s ability to use novel data sources for extracting meaningful public health information from unstructured data sources was considered a major advantage, that could supplement traditional disease surveillance.

You know, our traditional approaches, analysis, regression, so on work with structured data and you can work with even large amounts of structured data[…] But when you’re talkin’ about data that’s coming, and, again, you can think of all these sorts of unstructured data from large numbers of people … Like, basically if you look at that raw data every second of your day... There’s a record for that. Um, you can’t analyze that with traditional methods, and you don’t necessarily want to, right? So, you need tools [like AI] that will take one individual’s data [and] turn it into something meaningful. And then you’ve got millions of people’s worth of data.

[Participant ID # 7].

One example pointed out by participants, HealthMap [11], uses a combination of official and unofficial online, unstructured data sources for disease surveillance (see Additional file 6).

In addition to making use of novel sources of data, participants pointed out that AI could allow us to perform disease surveillance in a more timely and ongoing fashion.

… looking at things like mortality data. We’ve had a very significant lag. It gets coded, it gets cleaned, it gets deposited at the provincial or national level. The data can be a year or two out of date. […] With some of these [AI] tools we are going to have the opportunity to really accelerate and for the first time have population-based data in real time.

[Participant ID # 9].

An example highlighted by respondents entailed using hospital triage data for real-time surveillance (see Additional file 6).

Better use of large, linked datasets for both disease surveillance and exploratory analyses is another opportunity identified by participants.

It’s difficult to think of the dataset that wouldn’t potentially have relevance to public health. And this is the point when we start doing these big data fusion exercises, you often find that the most interesting, informative dataset is one you’ve never thought of. You just threw it in there because you had access to it.

[Participant ID # 4].

Experts pointed to a platform being piloted that uses a sophisticated ontology to integrate administrative health data, health survey data, and electronic medical records data, while also automatically providing evidence-informed population health priorities, causal information, and applicable preventive interventions (see Additional file 6).

In a related sense, experts pointed out that AI may prove capable of sifting through big data to facilitate knowledge translation broadly, as well as the creation of guidelines (see Additional file 6).

Beyond improving existing disease surveillance systems, it was thought that AI might allow leapfrogging in places lacking traditional infrastructure. In so doing, AI could enable earlier and more effective control of burgeoning epidemics.

… there [are] a lot of places in the world where there is absolutely no public health infrastructure whatsoever and when things kick off there, that’s where you start to get your […] Ebola outbreaks or your Zika virus outbreaks. […] I picture [AI] as being […] an easy sort of tractable way to get surveillance into places where spillover events, where emerging infections are likely to kind of pop up and where they would otherwise generally go missed due to a lack of laboratories and infrastructure.

[Participant ID # 6].

### AI will improve public health interventions

Participants affirmed AI’s potential to advance public health interventions, including screening for diseases where it may sometimes already exceed human performance (see Additional file 6).

In addition to automating existing screening programs, AI may make entirely new forms of screening feasible.

I think the cost of these technologies is going to continue to come down. So, I don’t want to get too sci-fi, but you could imagine a future where everyone’s got a device of some type. […] So cheap that the government actually provides everyone with a personal health monitor.

[Participant ID # 7].

Experts also thought that AI’s ability to better leverage real-time insights from big data may facilitate nearly instantaneous design and enactment of preventive interventions, creating a much nimbler loop of learning and action.

I think [AI’s] also going to enable us to design and implement interventions in real-time. So, to be able to do like […] internet companies[, who] will do A/B testing on the color of that button that you got right there and see which one people are more responsive to. We can start to think about [similar real-time interventions] in public health. Uh, can we go into supermarkets and change the way in which signs around vegetables are presented, and monitor cash register data in real-time. So, you can design health promotion interventions in real time.

[Participant ID # 7].

Some interviewees went on to suggest that these real-time AI interventions could be used for more personalized health promotion, particularly through using social media information.

You’ll probably be accused of nanny state-type intervention, but, you know, every time you see someone eating a cheeseburger and French fries on their [social media platform] you say, ‘Hey, [ …] how about some vegetables?’

[Participant ID # 4].

Rather than simply personalizing health promotion, it was proposed that we might apply social networking models to target public health messaging for maximal impact.

… machine learning plays a critical role in sort of identifying where your opportunities are to influence and, you know, if I look at who’s following whom on [social media], I actually only need to influence four people and now I can influence 400,000.

[Participant ID # 4].

However, not all respondents thought there was great potential for more adaptive and targeted health promotion interventions (see Additional file 6).

### What is AI for?

Beyond leveraging big data and improving interventions, experts were in less agreement regarding AI’s applications. Some thought that AI’s greatest potential lies in causal inference and hypothesis generation.

What are the factors that contribute to drowning in [the] beach? [You] look for similarities, patterns, trends that were not anything that would have been [previously] comprehended because people would focus on, you know, they couldn’t swim or they didn’t have life jackets. But it may be that they all have congenital heart disease. Again, not likely […], but you’re getting my drift.

[Participant ID # 9].

In contrast, other interviewees thought that AI has little potential for identifying novel causes.

… we’re not going to discover new risk factors. I don’t believe that. I don’t believe these methods are going to reveal risks for sub-populations that we don’t already know.

[Participant ID # 12].

Indeed, the interpretability of machine learning algorithms is widely considered to be an issue. Many AI methods, especially deep learning, are considered inscrutable. However, some participants thought that these issues could be overcome.

I mean the two hottest research areas in machine learning and AI right now are explainability and causal reasoning. So, I think there is a huge demand from many, many, many perspectives to be better able to explain those things and to be, um, you know, to be able to learn causal association. Or causal, you know factors. […] My sense is we’ll get better at it. I don’t know if it’ll ever get perfect with the really, truly data-driven methods, but I think we’ll see big improvements in the next few years.

[Participant ID # 3].

Despite the potential for increasingly interpretable AI, it is more commonly applied to predictive analytics. Some interviewees thought that this will be an important application for public health (see Additional file 6). However, even for prediction applications experts point to difficulties in knowing when to use AI approaches, which are often considered much more flexible than standard regression techniques.

… the question then becomes is the extra information that comes from loosening [the model] up actually valuable and useful? That’s what we need to still figure out. We don’t have a good handle on that yet. But I think we will. […] And then we can refine our efforts at focusing on those specific places instead of just saying, oh we need to just […] neural network everything.

[Participant ID # 12].

### Limited capacity

Many respondents identified barriers to applying AI in public health, such as limited availability of AI expertise and a lack of leadership on it in public health.

Right now, it’s hard to see that drive coming from within public health and […] it’s hard to see it […] coming from the AI community because there’s so many more low-hanging fruits that are […] competing for their attention right now.

[Participant ID # 3].

It was thought that cross-training of those with public health expertise, including medical students who become public health physicians, would be helpful.

They need to be exposed to, here’s really basic high-level intuition of how machine learning works. Here’s what the tools can do for you as a physician whether you become a radiologist or public health physician. And here are some examples of how they’re applied. And that’s really all you can do.

[Participant ID # 4].

Experts also thought that hiring staff with dedicated AI expertise was important, but noted it was difficult to compete with industry and more healthcare-oriented sectors (see Additional file 6).

… if you need talent to do stuff like this, you’re competing with industry offering […] eye watering salaries.

[Participant ID # 4].

And while participants had found significant interest in AI applications for public health from decision-makers, it was difficult to obtain the financial support needed to innovate.

All of that sort of technical and brain power stuff that’s been tough for me to get, um, because I have no money. I have no money or no resources, so I’m trying to build relationships to get us to do that. So, the government in themselves have not been able to get me there. They like the concepts. Everybody agrees with the concepts. All the big people agree with the concepts. It’s delivering it.

[Participant ID # 9].

### Lack of quality data

Experts frequently emphasized that most AI approaches are predicated on access to clean, high-quality data, which can be difficult to find for health applications.

… the fuel for artificial intelligence is data and the state of data, of health-related data right now is... It’s pretty dismal.

[Participant ID # 13].

For example, while there has been significant interest in using AI to leverage clinical notes in electronic medical records (EMRs) for disease surveillance, the quality of this data is a concern.

I must say I’m not overly optimistic of the value of [applying natural language processing to EMRs]. […] I’m not [sure] what you get out of there that’s not already in structured format [and] that you can do consistently in a repeatable manner. […] It’s going to be highly variable depending on the EMR. […] I think it’s a value but […] I think it’s a big heavy job to do it across multiple EMRs and the [whole region] and do a good job of it.

[Participant ID # 3].

Increased standardization was suggested to improve the usability of health data and the potential for linkage among various datasets (see Additional file 6).

Privacy concerns were acknowledged as another concern when obtaining data for public health purposes.

Yeah, some hospitals, their privacy officers are just so concerned. There’s such a fear now of, you know, the potential for re-identification and privacy. Sometimes it’s about the time and resources for them to come on. It’s about risk and liability and it’s really unfortunate. We’ve done like just so much work. We’ve spent so many hours. We’ve had legal counsel involved. Uh, just to overcome privacy concerns. Yeah, it’s quite an issue.

[Participant ID # 8].

Some thought that privacy concerns among administrators, lawmakers, and scientists may not realistically reflect citizens’ concerns.

… the public are a lot savvier than we give them credit for and people are very good at distinguishing between corporations use of data for […] targeting advertising […] versus […] medical and scientific use of data to improve peoples’ health. […] We often are afraid that we’re going to be painted with the same brush that people paint […] [social media companies or political consulting firms, thinking that] if we use anything to do with that and if we even say the word data people are going to get really angry.

[Participant ID # 6].

Finally, many datasets of interest to public health are owned by private companies, making their use difficult.

[This computer’s company’s] got data, [that wearable company’s] got data, there’s other firms that have... [This sporting goods company’s] got their systems, right. So, everyone’s got their systems. And there’s really no way for public health, if we felt we wanted to use that for surveillance purposes, we would have to go out and negotiate with each one.

[Participant ID # 7]

### Bias must be controlled

Participants were concerned about the potential for AI to propagate social and cognitive biases that can become part of the datasets used by AI algorithms for training.

… data and algorithms just kind of absorb and amplify the biases that we already have.

[Participant ID # 10].

It was also a concern that datasets used for AI may have selection bias, meaning participants that are not representative of the population as a whole. They also noted that there may have been inadequate attention paid to this issue when applying AI.

Definitely [traditional epidemiological] studies have the potential for those biases, although we do a lot to try and minimize them or at least them and quantify the impact. And so, I think the difference is we’re not talking about this with AI. I haven’t seen any discussion of oh, we should quantify the impact of the selection bias in our, you know, neural network. Or I’ve seen much less of that.

[Participant ID # 12].

Furthermore, it was pointed out that much of the data used with AI is generated as a by-product or for purposes unrelated to proposed applications. This can make it more difficult to understand potential biases and errors in measurement of variables of interest (see Additional file 6). Despite these concerns, some interviewees pointed out the potential for AI to remove bias from judgments.

… [AI] removes that human element, which in some ways is quite good [even though] some people don’t like it. I think there’s a huge benefit of having that human element and that potential bias removed from the […] actual actions.

[Participant ID # 9].

It was suggested that AI approaches could be trained to reduce bias, and that while bias is a risk it could be overcome with more research (see Additional file 6).

I think it’s easy for people to say well that data is garbage and that is garbage. I think we need to get more nuanced to say when, what measurement error counts when and where?

[Participant ID # 12].

### Uncertain impact on inequity

Respondents were worried that the use of AI and novel complex data sources to better target interventions in public health (sometimes referred to as precision public health) [25] could worsen health inequity, both within and between countries, based on ability to afford the necessary technologies.

… one of the huge concerns that I have around so-called precision medicine, precision public health, is it just strikes me that these are inherently inequity exacerbating […] for a variety of reasons. […] Who’s gonna be within a health system that’s got the capacity to develop these things and produce products that are used for … You know, it ain’t going to be low- middle-income countries. It’s going to be high-income countries and it’s going to be specific communities, you know, entities, economic strata, within that.

[Participant ID # 1].

Furthermore, there is the potential for selection bias in the data used for AI applications to further marginalize underrepresented populations (see Additional file 6).

[If your data is not representative of the population] you could be, you know, making things worse, consistently, systematically, for people in terms of what you’re recommending or detecting or not detecting.

[Participant ID # 3]

However, others proposed that the increased health information and service-accessibility enabled by AI may in fact reduce inequities.

… we know that better-educated, higher-income people tend to have healthier diets and access to better information […] So, [we may reduce inequity] if we’re able to create better personalized tools, […] like a voice interface that can give someone with a low reading level access to good nutritional information.

[Participant ID # 7].

### Hold the hype

Despite major advances, interviewees believed that AI is shrouded in hype, and that this could lead to it taking resources away from proven approaches.

That whole concept that, you know, this is what’s new, this is what’s getting hyped, this is what’s absolutely sexy and is starting to suck in policymakers, funders, etcetera. To the point where we take from where we should be investing because the historical track record indicates that it has produced good.

[Participant ID # 1].

They stressed that it is not so different from traditional statistical methods and is subject to similar limitations, which is often not fully appreciated.

You can think of AI as just […] the next set of tools in statistics. Yeah, we moved beyond t-tests and we have convolutional neural networks now but the basic principles […] are fundamentally the same …

[Participant ID # 4].

As such, participants thought that it should be emphasized that concerns regarding issues like bias apply equally to AI applications, as they always have in statistical applications to public health. It was posited that AI researchers have been mostly focused on developing advanced methods, while data generation and potential bias has been of less concern, leading to some of this confusion.

[The computer science] discipline has been focused on developing the method, not generating the data. So, and that’s not a criticism, that’s just what they do, and it’s great that they do. But when you’re in public health […] you have to focus on […] generating that data or understanding the pros and cons of different data for a public health application. We spend a lot of time, actually more time, on the dataset, data creation, data analysis, […] data interpretation, data integration than anything.

[Participant ID # 12]

Experts indicated that AI has been most impactful so far in commercial efforts, such as sales and marketing, where there is often higher tolerance for errors than in public health.

[A soda company brand] can maybe afford some level of error […] in what a machine spits out when it’s doing a marketing campaign to sell more [beverages.] [….] We don’t have that same luxury […] where we’re dealing with human life and human health. And so, I think that’s going to be a struggle for us because we’re going to need to have that higher threshold of accuracy and more confidence that [the] machine-created algorithm is going to be acceptable.

[Participant ID # 2]

There is also a concern that AI-based errors could be even more damaging than those of poorly performing human public health practitioners or doctors.

… if it were just one doctor making a mistake, then that is fine, […] I mean that’s not fine, but at least only one patient is harmed. But if an AI makes a mistake, then potentially tens of thousands of patients will […] be harmed.

[Participant ID # 13].

### Rigorous regulation required

Several respondents were concerned about the largely unregulated nature of AI and its use in public health.

… a lot of these tech groups operate under the same ethos that, [various companies in Silicon Valley do], which is ‘move fast and break things’. […] And, in [health], when you move fast and break things, lives are at risk. And so, a worry of mine is that some of these AI groups are going to potentially move too quickly.

[Participant ID # 14].

As such, most participants agreed that rigorous regulation is necessary. However, they were unsure of exactly what form this should take.

So, somehow, we almost need like [a Food and Drug Administration] for artificial intelligence, that will regulate constant evaluation of the AI tools that we are incorporating into practice.

[Participant ID # 13].

## Discussion

Our findings from this qualitative study can serve as a preliminary roadmap of high-level issues for decision-making about AI in public health. Our results also support investigating multiple applications of AI, broadly including using AI to gain better insights from data and to develop improved public health interventions.

### New or improved information gathering

Participants were optimistic about the potential for AI-based applications to incorporate different types of unstructured data into disease surveillance, to do this in a more continuous and timely fashion, and to better leverage large, linked databases. Existing examples highlighted by participants include using natural language processing to analyze emergency room triage data in real-time for anomalies, monitoring social media and news reports for emerging infectious diseases worldwide [11], and using ontologies to integrate public health-relevant data from numerous sources and identify evidence-informed intervention recommendations (e.g. PopHR) [26]. Another real-world example is the use of individual-level online search data to identify foodborne illness and prioritize restaurant inspections, which may be superior to traditional approaches [27]. More recently, BlueDot, a private company, used approaches similar to HealthMap (but also integrating proprietary airline ticketing data) to identify the Covid-19 outbreak before the World Health Organization [28], and was similarly successful in predicting the spread of Zika virus [29]. Our findings are consistent with an evolution of opportunities previously identified in public health informatics, which has progressed to integrating increasing amounts of data with less latency, permitting timelier action [30]. While promising, the identified applications using AI to leverage big data have yet to be widely used in practice, and further research is needed to assess their effectiveness and feasibility compared to existing approaches. Furthermore, most suggested applications would only complement existing public health indicators. Given that AI may enable entirely new approaches in public health, ongoing creative thinking could help to maximize its benefits. In particular, the combination of AI and big data may allow novel and more precise characterizations of the social determinants and their impacts on health [17].

### New or improved public health interventions

Opportunities for improved public health interventions were also outlined, such as automated and novel forms of screening for diseases, adaptive and personalized health promotion, and leveraging social networks for health promotion. As suggested by participants, certain forms of screening, including for cervical precancer, can already be performed with greater accuracy using AI [31]. Novel forms of screening have also begun, such as using machine learning to screen for atrial fibrillation with Apple watches, which was recently approved by the FDA [32]. However, unless such devices were proven to be cost-effective and ethical, they are unlikely to be useful in public health practice. Other expert suggestions included using AI to inform health promotion interventions in real-time, such as using transaction data to inform vegetable signage placement in grocery stores. This would be a type of “nudge”, or intervention that tailors the presentation of information to encourage beneficial behaviours [33]. Early systematic reviews demonstrate efficacy of such interventions in certain contexts [34]; however, there are ethical concerns regarding their use [33] and few thus far have used AI. The potential for using social media and more targeted health promotion approaches, some of which apply AI, appears promising in early reviews [35–37]. For example, AI-based chatbots have been used successfully as weight-loss coaches and to deliver cognitive behavioural therapy [38, 39]. Lastly, experts mentioned the potential for AI to leverage social network structure to increase the effectiveness of health promotion interventions. There is initial work in this area, including a group in California developing RECONNECT, an algorithm that connects individuals with friends who are likely to increase the chance of positive behaviour change [40]. Overall, the potential for AI to improve public health interventions is promising, as suggested by experts, but similarly to the use of AI for analyzing big data, these tools have rarely been incorporated in practice and need further study.

### Role in inference and prediction

Respondents offered somewhat divergent views regarding AI’s role in causal inference and prediction, which both inform public health practice. Causal inference in epidemiology is necessary for evidence-informed public health interventions. For example, Sir Austin Bradford Hill’s causal criteria were used to inform identification of the causal link between smoking and lung cancer, which has led to decades of successful preventive interventions [41]. An initial step towards causal inference is hypothesis generation, which machine learning algorithms such as random forest can contribute to by identifying “important” variables for predicting specific health outcomes in training datasets [42]. While AI has had limited use in more formal causal analysis, it is now being used for this purpose in targeted learning, an alternative that may outperform propensity score methods [43]. Outside of the specific algorithms that can be used for exploratory analyses and targeted learning, there seems to be limited applicability of AI for inference. However, as identified by participants, this is a very active area of research with new methods for interpreting algorithms under development [44]. Predictive algorithms can also inform public health practice by anticipating future disease burdens, directing policy, and targeting preventive interventions towards the highest-risk groups. For example, population-level predictive models have been developed to inform policy around dementia, diabetes, and cardiovascular diseases [45–47]. Despite substantial optimism about AI-based prediction, recent systematic reviews found no improvement in machine learning-based clinical prediction models generally [48], or for chronic diseases [49], when compared to logistic regression. However, it was acknowledged that most models only incorporated a small number of simple predictors, which might not fully leverage machine learning methods. Furthermore, a scoping review of machine learning-based prediction applications in population health found infrequent use of novel and large data sources, which may have hindered their performance [50]. Additionally, there was limited adherence to guidelines, which makes robust comparisons challenging. Overall, experts were divided on the utility of AI for inference in public health, with some stating that it would be unlikely to help identify novel risk factors. More fundamental research into methods development will be necessary to clarify AI’s applicability to causal inference. In the meantime, further studies applying amenable AI methods [16] to exploratory analyses in public health could help ascertain its utility for risk factor discovery. Finally, additional studies comparing machine learning approaches to traditional methods for prediction that use big data and adhere to guidelines are necessary to further clarify when AI might offer improvements.

### Barriers: capacity and data

Barriers to the adoption of AI in public health must be addressed before useful applications can be widely deployed. Key issues identified were a lack of leadership, available expertise, and funding to pursue AI approaches. Experts suggested that teaching high-level machine learning concepts to public health practitioners could be helpful in catalyzing AI initiatives. Interestingly, such an initiative has begun in Canada, scheduled to start in the summer of 2021 [51]. Stakeholders could also consider targeted funding for development of AI initiatives in public health, including programs for dual-trained AI and public health practitioners who can work productively with AI experts. Another concern is limited access to high-quality data. Experts highlighted numerous data sources of interest to public health, such as electronic medical records, administrative databases, health surveys, social media, and news reports with widely differing formats and standards. The lack of standardization (e.g. widely differing EMR software) and inconsistencies in data entry (e.g. in free-form clinical notes) make linkage of datasets and deployment of AI methods challenging [52]. This could be alleviated by adopting common data standards, alongside legislation that supports greater integration and access to relevant data. An initiative addressing some of these issues is underway in Canada, the Strategy for Patient-Oriented Research (SPOR), which aims to accelerate efforts to harmonize, link, and reduce barriers to access for a variety of governmental health and social datasets [53]. The adoption of standard EMRs and public health information systems across jurisdictions, that also encourage machine-readable data entry, would be an additional helpful strategy. Another issue noted by experts is the difficulty in accessing proprietary data that would be useful for public health, such as wearable or grocery transaction data. The success of BlueDot’s disease surveillance initiatives [28], using private airline data, and FINDER’s foodborne illness tracing [27], which involved a partnership with Google, suggest the feasibility and value of pursuing public-private partnerships. Relevant retail transaction data has also been integrated into PopHR [26]. Such partnerships could be accelerated with greater financial and research investment. Similar barriers related to overall capacity and data quality have been previously highlighted by public health informaticians, who have had some success developing interdisciplinary training programs and improving data integration [54]. This is encouraging for future deployment of AI in public health. Finally, as noted by experts, privacy concerns can prevent access to important public health data and must be addressed. It was suggested that the public might be more supportive than anticipated of public health’s use of their data, which is supported by initial studies [55–57]. Future research should continue to evaluate the public’s appetite for use of their data for public health applications in local contexts and develop best practices for the use of novel data sources. Policymakers and practitioners should follow these developments to inform improvements in data infrastructures and surveillance systems.

### Bias and impact on health equity

Many risks surrounding AI remain to be overcome. Experts were concerned about propagation of societal and cognitive biases, as well as intensification of information and selection bias. For example, racial bias was recently found in an AI algorithm designed to help guide referrals and future healthcare decisions [58]. The algorithm was biased towards predicting lower health needs for Black patients, because it used healthcare costs as a proxy for need. Less money had been spent on Black patients with the same level of need in its training data. Left unchecked, similar biases could lead to inappropriate allocation of services and preventive interventions in a public health setting. Furthermore, selection bias can have an impact when groups are not included in training data, resulting in suboptimal performance. This was highlighted recently among cardiovascular prediction models trained almost exclusively on White people [59]. Finally, measurement error can reduce the accuracy of AI models, as was seen with Google Flu Trends when changes in Google’s search algorithms and spurious media events changed what their algorithms were measuring [60]. Efforts to reduce the effects of these biases on AI applications include the adoption of big data standards, the use of algorithms to detect and eliminate bias, and greater efforts to be inclusive of the population as a whole during data collection [61]. Ongoing research is needed to improve understanding of the effects of these biases, implement strategies to adapt to their presence, and ascertain the suitability of new data-sources for use in public health. Furthermore, AI’s effects on inequities must also be evaluated continually. Ideally, as suggested by participants, AI investments should focus on areas likely to reduce inequities, including improved access to public health services and health information. This could include examples previously identified, such as an automated weight-loss coach [38] and the use of novel data sources to expand disease surveillance globally [11].

### Hype and regulation

Experts were concerned about hype surrounding AI. In addition to statistical limitations, they noted that the implementation of AI in public health will be more difficult than in industry, where the stakes are often lower. Consequently, the limitations of AI in public health contexts should be emphasized during related training. Furthermore, given a lack of evidence from implementation of AI in public health, it is uncertain to what degree AI techniques might improve upon public health actions that are already heavily data informed. Keeping these caveats in mind, it is essential that practitioners not significantly disinvest from traditional public health practice in their enthusiasm to adopt AI.

Our findings suggest that AI should be regulated; however, further work is needed to determine how and by whom. The Federal Drug Administration has recently proposed a regulatory framework for AI-based software as a medical device, that would uniquely permit continuous improvement and learning of approved algorithms [62]. While primarily aimed at healthcare, this is an important step that will inform regulation in public health. There is also a need for practice guidelines for the use of AI tools to inform both regulators and public health decision-makers. The GRADE approach to guideline development, particularly Evidence to Decision (EtD) frameworks [63], could be useful for systematically and transparently appraising not only the health benefits and harms of AI tools but also key considerations in the evaluation of AI including cost-effectiveness, equity, acceptability, and feasibility [64]. By applying the EtD framework to public health guidance around the use of AI, we could increase the chance that AI interventions truly have a net benefit to population health. The application of EtD frameworks would be based on additional work to identify best practices in the use of AI methods in health research. Finally, stakeholders must collectively decide which organizations would use these guidelines to regulate the use of AI in public health.

### Disruptive innovation

Viewing AI as a disruptive technology, our findings are consistent with disruptive innovation theory [65]. This theory describes a propensity for new technologies to take root in either market-segments that are over-served by mainstream performance capabilities or new markets made possible by innovations. Eventually, an innovation’s performance may improve to a point where it can serve mainstream needs. For example, personal computers were a disruptive technology that opened a new market by providing a lower-cost, lower-performance alternative to mainframe computers and minicomputers [66]. Eventually, their performance improved to the point that they could meet almost all market-segment needs. While applications of the disruptive innovation theory outside of private industry are imperfect, our findings highlight initial opportunities for AI in public health that are analogous to new markets [67], such as novel forms of disease surveillance and health promotion. These activities were previously impossible because leveraging big and unstructured data in real-time is not feasible for humans and standard statistical approaches. Therefore, these “new market” applications may be among the first widely used AI tools in public health. Conversely, respondents characterized core public health activities as requiring high-performance, including minimal bias, attention to health equity, and high accuracy, due to the sector’s impact on people’s health. These requirements explain experts’ concern about hype and predict that core public health will be more difficult to disrupt than the mainstream of other markets. For example, market segments interested in activities like targeted advertising of consumer products and movie recommendations may find that their needs are already met by the performance levels of current AI tools. The disruptive innovation perspective also suggests that public health organizations may be able to accelerate adoption of AI by establishing semi-autonomous innovation units [66]. This is analogous to the approach taken by the city of Chicago with their Department of Innovation and Technology, which helped the city’s public health department become a leader in the application of AI [68]. In jurisdictions without sufficient resources to consider this at the local or regional-level, such a strategy could be considered by state/provincial or national bodies. In the near-term, AI is likely to be restricted to use in new (previously impossible) public health domains with strict oversight. As AI’s performance improves, it may be applied to progressively more core public health needs.

### Strengths and limitations

To our knowledge, this is the first study to systematically and broadly assess experts’ appraisal of the implications of AI for public health practice. We used a credible qualitative study design that revealed diverse perspectives from multiple disciplines, countries, and settings.

Our study was limited by a small sample size and non-probability sampling. Therefore, in the context of the broad area of inquiry, our results are unlikely to capture all possible implications of AI for public health practice. However, this was not our intention. Additionally, our sample had a smaller number of female participants, which may reflect a lack of diversity in AI-research and industry [69]. We also recruited relatively few AI-focused researchers. Given that all invitees who refused to participate were in the AI field, this may reflect the lack of capacity identified in our study.

## Conclusions

Our results highlight that AI holds promise for improving public health practice; however, many barriers remain, and risks need to be better characterized. Experts emphasized the potential for AI to improve disease surveillance and health promotion interventions, which should be the focus of further research and evaluative studies. To successfully implement AI, initiatives increasing AI expertise and funding for public health are necessary. Public health policy innovations should improve the standardization, integration, and availability of relevant high-quality data. Further research is also needed to determine the best use-cases of AI, how to mitigate bias, and how to ensure a positive impact on health equity. In the meantime, training initiatives for AI-practitioners in public health should emphasize the limitations of AI, in order to combat hype. Finally, ongoing research and collaboration is needed to better regulate AI, steering it towards truly benefiting population health. As one respondent phrased it: “AI’s gonna have an impact on everything in society, so it has to have an impact on public health”. As this impact comes, it is up to the public health community whether we will be ahead of, or behind, the curve.

## Supplementary Information

**Additional file 1.** COREQ (COnsolidated criteria for REporting Qualitative research) Checklist: COREQ Checklist.

**Additional file 2.** Artificial Intelligence and Public Health: Interview Guide: Interview Guide.

**Additional file 3.** Description of Interviewers.

**Additional file 4.** Detailed Coding Information.

**Additional file 5.** Specific Participant Characteristics.

**Additional file 6.** Supporting Participant Quotations.

## Data Availability

The datasets generated and analysed during the current study cannot be made available due to the richness of qualitative data, the limited number of experts in this field, and the corresponding risk of re-identification of participants.

## Abbreviations

AI: Artificial intelligence
COREQ: Consolidated criteria for reporting qualitative research
EMR: Electronic Medical Record
NGO: Non-governmental organization
EtD: Evidence to Decision
