# Supplementary material for: “AI’s gonna have an impact on everything in society, so it has to have an impact on public health”: a fundamental qualitative descriptive study of the implications of artificial intelligence for public health
Source: BMC Public Health. 2021 Jan 6;21:40. doi: 10.1186/s12889-020-10030-x (PMC7787411; doi:10.1186/s12889-020-10030-x)
Supplement: Supplementary file 2 — Additional file 2. Artificial Intelligence and Public Health: Interview Guide: Interview Guide. [file 12889_2020_10030_MOESM2_ESM.docx]

**Artificial Intelligence and Public Health**

Interview Guide

**A. Participant Information**

| **Participant no.:** |  |
| --- | --- |
| **Participant name and contact information:** | Name:  Email/Telephone: |
| **Location:** |  |
| **Date:** |  |
| **Interviewer/Note-taker:** |  |
| **Recorder interview no.:** |  |

**B. Interviewer Checklist**

***For in person interviews:***

- Confirm the date, time and location of the meeting, send a reminder to the participant before the meeting

- Ascertain allotted interview time acceptable to participant, consider excluding some questions if necessary

- Send participant primers on AI and public health as applicable ahead of time

- Print copy of Interview Guide, take notes (point-form preferred) in the spaces provided

- Prepare additional paper to take notes if needed

- Prepare audio recorder, test the recorder before each interview

- Set timer at the beginning of interviewer to aid in prioritization of questions

***For interviews by telephone or Skype:***

- Confirm the date and time of the call and send a reminder to the participant before the meeting

- Make sure you have the land line phone number or Skype contact

- Ascertain allotted interview time acceptable to participant, consider excluding some questions if necessary

- Send participant primers on AI and public health as applicable ahead of time

- Print copy of Interview Guide, take notes (point-form preferred) in the spaces provided

- Prepare additional paper to take notes if needed

- Prepare audio recorder, test the recorder before each interview

- Set timer at the beginning of interviewer to aid in prioritization of questions

**C. Introduction and Consent Statement**

**Say**: Thank you very much for agreeing to participate in this interview. We are asking artificial intelligence (AI) experts about current and future impacts on the field of public health. The field of public health is engaged in preventing disease and promoting health at the population level.

We will use your input to better understand emerging AI technologies as they relate to public health and to help guide future AI adoption, regulation, and research within the field of public health.

**Participant Consent Statement:**

**Say**: The research study has been reviewed by the Hamilton Integrated Research Ethics Board (HiREB). With your permission, the session will be recorded on tape for transcription and erased after the transcription has occurred. Transcribed data will be analyzed and coded into themes, and destroyed at the end of the study period and publication. De-identified coded themes will be destroyed after a period of 10 years. You may also withdraw your responses from the interview at any time.

Do you agree for the data collected in the study to be used anonymously in publication?

☐ Yes ☐ No Notes:

Do you also agree to have the interview recorded?

☐ Yes ☐ No Notes:

**Ask**: Do you have any questions before we proceed?

**D. Public Health Primer**

**Ask**: Do you have any questions regarding public health focus areas and/or current standards of practice in Ontario?

☐ Yes ☐ No Notes: (answer queries as necessary and document)

**Say:** Public health includes actions at a community or population level to protect people from diseases or improve their health. Many public health activities relate to historical responsibilities such as hygiene, food safety, and vaccines. In thinking about the implications of AI on public health, don't limit your thinking to these activities. Instead try to think about the broad definition of protecting people from diseases and improving their health.

**E. Questions**

*The interview will be semi-structured and therefore follow-up questions to probe further into interesting issues and to clarify statements will be posed at the discretion of the interviewer. If a question in the sequence has been addressed by earlier dialogue then these can be skipped at the discretion of the interviewer.*

*General*

1. In your opinion, what will be the most significant impacts of AI on Public Health practice?

Possible probes:

- Both positive and negative?
- In the short and long-term?

|  |
| --- |

*Scenarios*

Opioids

2. Public health is currently grappling with an opioid crisis. Opioids have been increasingly prescribed and consumed for a variety of reasons over the past 20 years. There are now more deaths caused by opioid overdoses than motor vehicle accidents in Canada. Public health practitioners are trying to respond using a variety of strategies including: surveillance, education, harm reduction, and working with law enforcement to decrease opioid deaths. How do you think AI-related work could impact this public health crisis?

Core related area(s) of public health: health equity

|  |
| --- |

Pandemic Preparedness

3. A core function of public health is to protect the population against the spread of pandemic diseases. Many communicable diseases have decreased dramatically over the past 100 years with the advent of vaccinations and routine sanitation practices, however, certain diseases continue to emerge and re-emerge as public health threats. Recent examples include: Zika virus, Ebola virus, and H1N1 influenza virus. How do you think AI work could improve public health’s capacity to detect and mitigate pandemic threats?

Core related area(s) of public health: health protection, surveillance, health system capacity building

|  |
| --- |

Sugar consumption and its relationship to obesity, diabetes, and other chronic diseases

4. Obesity is a rising health concern that has been linked to increased sugar consumption among other societal changes. Public health has often focused on educating the public regarding sugar-consumption, however, more recently is acknowledging that education has its limitations due to structures in society such as corporate interests and inequalities that undermine people’s ability to make healthy food choices. How do you think AI work could advance the public health response to sugar-consumption and obesity?

Core related area(s) of public health: health promotion

|  |
| --- |

5. Final comments/additions

**F. End of Interview**

**Say:** Thank you very much for your participation.

*Additional Questions*

**Health Equity**

1. Currently there are vast inequities in health outcomes and access to healthcare in Canada. Within the city of Hamilton, for example, there is over a 20-year gap in life expectancy between some neighbourhoods. How do you think AI developments could affect our ability to reduce these inequities?

|  |
| --- |

2. How do you think AI will affect the future of employment and income inequality, and as a result public health practice?

|  |
| --- |

**Health Services**

3. Healthcare is one part of what makes people healthy; in terms of AI, what are the most important ways you think healthcare will be impacted?

|  |
| --- |

4. With advances involving low cost sensors, the internet of things, and AI, how much of healthcare might be transferred into the home with no necessary involvement of human professionals? Could these advances be accessible to all? Should they be publically funded?

|  |
| --- |

5. Mounting evidence has demonstrated that the traditional yearly doctor check-up is largely ineffective in preventing disease. More recently, the proliferation of wearable biometric devices, social media, and connected smart home applications have raised hope for new approaches to disease surveillance, prediction, and prevention. What opportunities and risks do you foresee in applications of AI to personalized, preventative medicine?

|  |
| --- |

**Health Promotion**

6. In your opinion, what are the reasonable advances we could expect in social applications of AI to health? For example, one company has created ‘Woebot’, a chatbot that engages users in Cognitive Behavioural Therapy (CBT). In the extreme, movies like ‘Her’ forecast a future where human beings might even enter intimate relationships with their AI companions. In particular, could these developments be useful for promoting healthy behaviour and understanding barriers to health?

|  |
| --- |

**Monitoring, evaluation, and analysis of health status**

7. Canada’s adoption of electronic medical records (EMRs) has been fragmented, resulting in very different systems neighbouring health units with no capacity for information exchange. What’s worse, many of these EMRs store information in mostly natural language format, making it difficult to extract much of the useful data for analysis. Would a more integrated system be beneficial? How feasible is this? How optimistic could we be that the natural language data could be reliably extracted by AI in the near future? Could AI reasonably assist us in bridging together currently incompatible software?

|  |
| --- |

**Risks of AI**

8. As we attempt to leverage various data sources for prevention and identification of disease, what privacy issues do you anticipate and how, if at all, might these be overcome? What other risks do you foresee in both the short and long-term, including issues of safety, human agency, and identity?

|  |
| --- |

**Public Health Research**

9. Currently much of public health research relies on humans accessing and interpreting data that has been sitting in various repositories for extended periods of time. Additionally, research often involves a very time-consuming systematic review of existing scientific literature. Do you think advancements in AI might allow much of this research to become automated? Furthermore, what opportunities might there be in identifying environmental risks, previously unknown causes of disease, and in predicting natural disasters? How about knowledge translation?

|  |
| --- |

**Public Health Policy and Administration**

10. Could AI assist public health doctors in developing public health policy, prioritizing public health and other healthcare expenditures, and identifying inefficiencies?

|  |
| --- |

**Public Health Enforcement**

11. How might AI influence our ability to enforce public health regulations? How about monitoring physician diagnostic and treatment outcomes?

|  |
| --- |
