# Supplementary material for: “AI’s gonna have an impact on everything in society, so it has to have an impact on public health”: a fundamental qualitative descriptive study of the implications of artificial intelligence for public health
Source: BMC Public Health. 2021 Jan 6;21:40. doi: 10.1186/s12889-020-10030-x (PMC7787411; doi:10.1186/s12889-020-10030-x)
Supplement: Supplementary file 3 — Additional file 3. Description of Interviewers. [file 12889_2020_10030_MOESM3_ESM.docx]

**Additional File 3.** Description of Interviewers.

All interviews were conducted by JDM and TP. JDM was working as a public health and preventive medicine resident and Master of Public Health candidate during the study. TP was working as a public health and preventive medicine resident, family physician, public health physician, and PhD candidate throughout the study. Both interviewers identified as male. There was a mix of pre-established and novel relationships between interviewers and interviewees. JDM reviewed “Qualitative Methods for Health Research”^21^ prior to conducting interviews and was trained by TP. TP had training in qualitative research methods from dedicated course work at the London School of Hygiene and Tropical Medicine in the MSc in epidemiology program, in addition to other extra coursework undertaken throughout his prior four years of residency. He had also undertaken over 50 interviews as part of other qualitative research projects, including a study evaluating expert clinical guideline panels. TP supervised JDM throughout the study. Interviewees knew about the interviewers’ occupations and that they were pursuing this project as an extracurricular activity, out of interest. The interviewers’ occupational positions and research questions were reported to participants.
