# Supplementary material for: “AI’s gonna have an impact on everything in society, so it has to have an impact on public health”: a fundamental qualitative descriptive study of the implications of artificial intelligence for public health
Source: BMC Public Health. 2021 Jan 6;21:40. doi: 10.1186/s12889-020-10030-x (PMC7787411; doi:10.1186/s12889-020-10030-x)
Supplement: Supplementary file 6 — Additional file 6. Supporting Participant Quotations. [file 12889_2020_10030_MOESM6_ESM.docx]

**Additional File 6.** Supporting Participant Quotations.

**From Big Data to Big Insights**

…one of the really exciting opportunities is using AI […] for improving our surveillance programs. Particularly by leveraging some non-traditional sources of surveillance information.

[Participant ID # 6]

… it's this tool that’s just constantly scanning a whole bunch of online resources like newspapers in a bunch of different languages and health department notices, um, a couple of social media streams, just looking constantly looking for: do we see a blip? Do we see a pattern that's potentially suggestive of a disease event? So that's I think one of the, uh, the best developed systems out there. They're definitely leaders in the field.

[Participant ID #6]

… it's a system, a real-time system, where we receive triage data from [a large number of] hospitals all across [the region] here in our data centre 24 hours a day, 7 days a week and, um, we're using machine learning techniques on that, in real time, on that triage data to see if we can detect anomalies and potential threats to public health.

[Participant ID # 8]

It's kind of like precision public health. So, so when you're looking at a given population [you] can assess the characteristics of that population in terms of, you know, from the indicators. [There’s] age, sex all that kind of stuff and [socioeconomic status indicators] and, uh, predominant health conditions and then [it can] basically recommend […] interventions that are evidence-based [and] that address the most important determinants of health in that population.

[Participant ID # 3]

… it's imaginable that evidence to decision frameworks will be completed by machines […] That will still require human judgments. But also looking very far into the future, I mean the way that things are developing, it's not totally unreasonable that, you know, you would have recommendations produced through machine learning exercises.

[Participant ID # 15]

**AI Will Improve Public Health Interventions**

I think they're pretty close to the point where reading pap smears can actually just be replaced by the AI altogether.

[Participant ID # 7]

… fundamentally, we know that individually focused health promotion is limited in its population health impact. So, I don't see it transforming the way we do health promotion.

[Participant ID # 12]

**What is AI for?**

Now the predictive piece of AI is where I think that there's a lot of potential. The idea that we have a forward looking […] way to strategize in terms of chronic disease prevention and infectious disease. […] I think we'll do more of it and […] we'll have new analytic tools to do it.

[Participant ID # 12]

**Limited Capacity**

I mean, there's no business really in public health to be honest. But there certainly is in Radiology.

[Participant ID # 3]

**Lack of Quality Data**

So, number one is to work on standardizing the data right, um, going forward. So, you know, really thinking about a common data model. Again, this comes down to good data governance practices and interoperability practices.

[Participant ID # 10]

**Bias Must Be Controlled**

With AI for the most part we're actually talking about passive data collection. Data that's collected for other purposes, which may or may not have been designed for that application that we're using it for. And it may have measurement error in it.

[Participant ID # 12]

It takes out bias, right. I believe it takes bias away, you teach it to not have bias.

[Participant ID # 9]

**Uncertain Impact on Inequity**

… if you want a public health AI platform that is going to speak to resolving health inequities, you better make damn sure that everybody is represented in your training data that you're using, you can't be leaving out any sector of the population.

[Participant ID # 6]
